# Vibration Characteristics of Alumina–Steel Axially Functionally Graded Fluid-Conveying Pipes: A Physics-Based GITT and MLP Surrogate Study

**DOI:** 10.3390/ma19132745

**Published:** 2026-06-26

**Authors:** Lun Gao, Jijun Gu, Tianjin Guo, Shanshan Zhao, Junjie Li

**Affiliations:** 1 School of Safety Science and Engineering, Xinjiang Institute of Engineering, Urumqi 830023, China; gl07@xjie.edu.cn (L.G.); gtj@xjie.edu.cn (T.G.); shanshanzhao@xjie.edu.cn (S.Z.); ljj@xjie.edu.cn (J.L.); 2 College of Mechanical and Transportation Engineering, China University of Petroleum-Beijing, Beijing 102249, China

**Keywords:** functionally graded materials, vibration characteristics, Alumina–Steel, generalized integral transform technique, multi-layer perceptron, pipe-conveying fluid, sensitivity analysis

## Abstract

The vibration characteristics of clamped–clamped Alumina–Steel axially functionally graded (AFG) fluid-conveying Timoshenko pipes are investigated using a physics-based generalized integral transform technique (GITT) benchmark and a multi-layer perceptron (MLP) surrogate trained on GITT data. Parametric GITT sweeps over the power-law gradation index *k*, dimensionless flow velocity *u*, and aspect ratio L/D quantify how axial material gradation controls the first two natural frequencies ($\omega_{1}$, $\omega_{2}$) and the maximum vibration deflection ($y_{M}$): increasing *k* reduces $\omega_{1}$ and $\omega_{2}$; on *u*-sweeps at L/D=50, larger *k* also increases $y_{M}$ and lowers the critical flow velocity, whereas on L/D-sweeps at u=3.0, $y_{M}$ decreases with *k*. A feedforward MLP surrogate fitted to $N_{s}=336$ GITT samples via an interior block-wise train–test split and three independent networks with output-specific preprocessing achieves $R^{2}>0.99$ on held-out data, with maximum relative errors below 9%, and reproduces representative GITT parametric curves in overlay validation. After one-time offline training, MLP inference is orders of magnitude faster than online GITT runs, enabling large-scale global sensitivity analysis based on Sobol indices, SHAP values, and partial dependence plots; these identify *u* as the dominant influence on the modal responses, while SHAP ranks *k* first for $\omega_{2}$. The physics-based GITT and MLP surrogate workflow combines high-fidelity material–structure benchmarking with efficient metamodeling for design optimization, reliability assessment, and sensitivity-driven screening of Alumina–Steel AFG fluid-conveying pipes.

## 1. Introduction

Axially functionally graded (AFG) materials are characterized by continuous spatial variation of composition and microstructure, which leads to tailored distributions of effective properties. Owing to this design flexibility, AFG structures have found broad applications in reactor vessels, fusion systems, biomechanics, automotive, aerospace, civil, and marine engineering [1,2]. Consequently, the vibration and stability of AFG beams have been extensively investigated.

Early studies mainly focused on free and forced vibrations of AFG structures under different boundary and loading conditions. Representative contributions include semi-inverse solutions for AFG beams [3], moving-load-induced vibration [4,5], non-uniform cross-section effects [6], shell-type FG structures with elastic foundations [7], and vibrational characteristics of FG plates with general boundaries [8].

To capture rotary inertia and transverse shear deformation, many researchers adopted Timoshenko-based formulations. These works addressed tapered and non-uniform AFG beams [9,10,11,12], boundary-condition and solution-method effects [13,14,15], bi-directional and double-FG configurations [16,17], and coupled vibration-buckling responses under thermo-mechanical loading [18].

For pipes conveying fluid, substantial foundations were established in classical studies [19,20,21,22,23,24,25,26]. For instance, Liu et al. [27] recently investigated the aerodynamic flutter of blooie lines during gas drilling in mountainous areas, highlighting the severe vibration risks induced by sudden high-speed gas flow and blockages. Recent efforts have moved toward functionally graded pipes and more complex fluid–structure interactions. In linear stability analysis, Zhou et al. [28] clarified the roles of elastic-modulus and density gradients in flutter boundaries, while Tuo et al. [29] and Fu et al. [30] used generalized integral transform solutions for AFG pipes with single-phase and gas–liquid two-phase flow, respectively. Mao et al. [31] further investigated nonlinear dynamics of AFG Euler–Bernoulli pipes and reported gradient-induced non-trivial equilibria and response asymmetry.

Additional developments considered more involved excitation and coupling mechanisms. Jing et al. [32] analyzed parametric resonance of AFG pipes conveying pulsating flow. Within Timoshenko-based pipe models, Shao et al. [33] examined natural frequencies and critical velocities, and Sabahi and Saidi [34] studied nonlinear vibrations for two-phase internal flow. Other extensions include spinning multi-span FG pipes [35], FG-CNTRC pipes based on Timoshenko theory [36], size-dependent stability of axially graded micro-pipes [37], and nonlocal thermo-mechanical vibration of spinning FG nanotubes conveying fluid [38].

These studies demonstrate the critical roles of material gradation, flow regime, and multi-physics coupling in pipe dynamics. However, the current literature remains fragmented in structural assumptions, boundary settings, and solution strategies. Many AFG pipe studies rely on Euler–Bernoulli theory, whereas many Timoshenko-based studies target specific scenarios such as spinning systems, micro/nano scales, composite reinforcement, or pulsating/two-phase flow. In particular, a unified Timoshenko–GITT characterization that simultaneously addresses the coupled influence of *k*, *u*, and L/D on both modal frequencies and maximum deflection remains lacking for clamped–clamped Alumina–Steel AFG pipes.

High-fidelity methods such as GITT [29,30,39,40] provide reliable benchmark solutions, but each parametric case requires eigenfunction evaluation, coefficient integration, series truncation, and time-domain integration of a coupled ODE system. This computational burden becomes prohibitive for design optimization, Monte Carlo simulation, and global sensitivity analysis [41,42]. In recent years, machine-learning-based surrogate modeling has emerged as an effective strategy for replacing repeated expensive simulations in structural and vibration problems. Representative examples include residual MLP surrogates for band-gap optimization of periodic beams [43], ANN-based metamodels for structural response approximation [44,45], and physics-informed neural networks for fluid-conveying pipe vibration [46]. These studies indicate that data-driven models can preserve the accuracy of high-fidelity solvers while dramatically reducing online evaluation cost. However, to the authors’ knowledge, a dedicated MLP surrogate that maps (k,u,L/D) to $\left(\omega_{1},\omega_{2},y_{M}\right)$ for clamped–clamped AFG Timoshenko pipes, trained directly on GITT benchmark data and coupled with global sensitivity analysis, has not yet been reported.

Accordingly, the present study investigates the vibration characteristics of Alumina–Steel axially functionally graded fluid-conveying Timoshenko pipes and develops a physics-based GITT benchmark coupled with an MLP surrogate for rapid parametric evaluation and global sensitivity analysis. The novelty of this work lies in the systematic integration of a physics-based GITT benchmark with a data-driven MLP surrogate within a unified workflow, which—unlike prior studies that apply either GITT or machine learning in isolation—enables both high-fidelity material–structure characterization and real-time parametric evaluation. This integration is particularly significant for practical applications where thousands of parametric queries are needed for design optimization, reliability assessment, and sensitivity-driven material screening; the surrogate reduces the wall-clock time for $10^{4}$ evaluations from approximately 131 h (sequential GITT) to about 1.6 s (trained MLP), representing a computational speedup of five orders of magnitude. The main contributions are summarized as follows:

- A Timoshenko–GITT formulation based on Gu et al. [40] is extended to an Alumina–Steel AFG pipe under clamped–clamped boundary conditions, with systematic parametric studies on *k*, *u*, and L/D.
- A feedforward MLP surrogate model is developed to approximate the GITT input–output mapping. After an 80/20 interior block-wise train–test split of 336 GITT samples ($N_{\mathrm{tr}}=270$, $N_{\mathrm{te}}=66$), the surrogate achieves $R^{2}>0.99$ on the independent test set, with maximum relative errors of 8.90%, 2.25%, and 8.89% for $\omega_{1}$, $\omega_{2}$, and $y_{M}$, respectively.
- Global sensitivity analysis based on Sobol indices, SHAP values, and partial dependence plots is performed on the trained surrogate to quantify the dominant influence of each input parameter.
- A direct comparison between GITT and MLP is presented in terms of predictive accuracy and computational efficiency, showing that the GITT–MLP workflow delivers a speedup factor of $\eta \approx 2.9\times 10^{5}$ for $10^{4}$ parametric evaluations, thereby enabling real-time design optimization, Monte Carlo simulation, and sensitivity-driven screening that would be computationally prohibitive with repeated Fortran GITT runs alone.

The remainder of this paper is organized as follows. Section 2 presents the physical model and governing equations. Section 3 describes the GITT solution procedure. Section 4 introduces the MLP surrogate model. Section 5 reports the GITT parametric results and validates the trained MLP surrogate. Section 6 presents the global sensitivity analysis enabled by the surrogate. Section 7 draws the main conclusions.

## 2. GITT Model Description

### 2.1. Physical and Material Model

**Figure 1:**
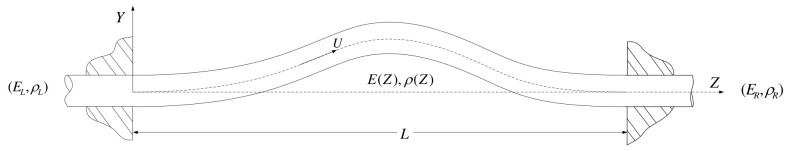
Alumina–Steel axially functionally graded Timoshenko pipe conveying fluid, subject to clamped–clamped boundary conditions.

The physical model of an Alumina–Steel axially functionally graded fluid-conveying pipe with fixed ends is illustrated in Figure 1, incorporating the effects of shear deformation and rotary inertia based on the Timoshenko beam hypothesis. At the supported ends, the transverse displacement and the slope of the transverse deflection are zero. Furthermore, the pipe is subjected to an internal fluid flow with a constant velocity, *U*.

**Figure 2:**
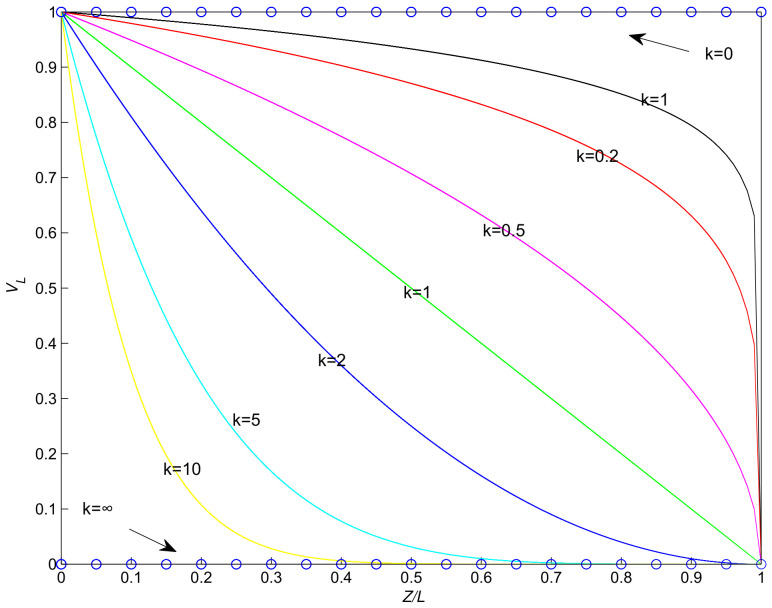
Axial variation of the volume fraction corresponding to the left-end material component (alumina, Al_2_O_3_).

This study assumes that the material properties of the AFG pipe are functions of the volume fractions of the constituents, varying along the axial direction. By the rule of mixture, the effective material properties *P* (i.e., Young’s modulus *E* and mass density ρ) are expressed as

(1) $$\begin{array}{cc} P & =P_{L}V_{L}+P_{R}V_{R},\\ V_{L}+V_{R} & =1,\\ V_{L} & ={\left(1-Z/L\right)}^{k},\\ E(Z) & =\left(E_{L}-E_{R}\right){\left(1-Z/L\right)}^{k}+E_{R},\\ \rho (Z) & =\left(\rho_{L}-\rho_{R}\right){\left(1-Z/L\right)}^{k}+\rho_{R}, \end{array}$$

where $P_{L}$ and $P_{R}$ represent the effective material properties at the left and right pipe ends, respectively, $V_{L}$ and $V_{R}$ are the corresponding volume fractions, and *k* is the power-law exponent. In the present study, the left end (z=0) is pure alumina (Al_2_O_3_) and the right end (z=1) is pure steel; therefore, the symbol $V_{L}$ denotes the local volume fraction of the alumina component throughout the paper. The variation in volume fraction $V_{L}$ along the axial direction corresponding to different power-law indices is illustrated in Figure 2.

### 2.2. Governing Equations and Nondimensionalization

Within the framework of Timoshenko beam theory, an axially functionally graded fluid-conveying pipe is considered in the present analysis. The pipe possesses the length *L*, cross-sectional area $A_{p}$, Young’s modulus $E_{p}\left(Z\right)$, moment of inertia $I_{p}$, mass per unit length $m_{p}=\rho_{p}A_{p}$, shear coefficient $\kappa_{s}$ and shear modulus $G_{p}\left(Z\right)$. For the fluid transported inside the pipe, the corresponding physical parameters include the moment of inertia $I_{f}$, mass per unit length $m_{f}$ and density $\rho_{f}$. Following the Timoshenko pipe GITT framework of Gu et al. [40] and the clamped–clamped integral-transform formulation of Gu et al. [39], the shear force *S* and bending moment *M* are introduced as

(2) $$S=G_{p}\left(Z\right)A_{p}\kappa_{s}\left(\frac{\partial Y}{\partial Z}-\theta \right)$$

(3) $$M=E_{p}\left(Z\right)I_{p}\frac{\partial \theta }{\partial Z}$$

The governing equilibrium equations for the AFG Timoshenko pipe are established as follows:

(4) $$\frac{\partial S}{\partial Z}=\rho_{p}A_{p}\frac{\partial^{2}Y}{\partial T^{2}}+\rho_{f}A_{f}a_{f}^{Y}$$

(5) $$\frac{\partial M}{\partial Z}+S=\left(\rho_{p}I_{p}+\rho_{f}I_{f}\right)\frac{\partial^{2}\theta }{\partial T^{2}}$$

Here, $A_{f}$ denotes the internal flow cross-sectional area and $a_{f}^{Y}$ denotes the fluid contribution to the transverse inertial acceleration of the pipe–fluid system [39]. By inserting the expressions for shear force *S* and bending moment *M* into the equilibrium equations, one obtains the governing differential equations for the transverse displacement Y(Z,T) and the rotation angle θ(Z,T), namely

(6) $$\begin{array}{c} \left(\rho \left(Z\right)A_{p}+m_{f}\right)\frac{\partial^{2}Y}{\partial T^{2}}+2m_{f}U\frac{\partial^{2}Y}{\partial Z\partial T}+m_{f}U^{2}\frac{\partial^{2}Y}{\partial Z^{2}}\\ -\left[\frac{\partial G_{p}\left(Z\right)}{\partial Z}A_{p}\kappa_{s}\left(\frac{\partial Y}{\partial Z}-\theta \right)+G_{p}\left(Z\right)A_{p}\kappa_{s}\left(\frac{\partial^{2}Y}{\partial Z^{2}}-\frac{\partial \theta }{\partial Z}\right)\right]=0 \end{array}$$

(7) $$\begin{array}{cc} \; & \left(\rho \left(Z\right)I_{p}+\rho_{f}I_{f}\right)\frac{\partial^{2}\theta }{\partial T^{2}}-\left[\frac{\partial E_{p}\left(Z\right)}{\partial Z}I_{p}\frac{\partial \theta }{\partial Z}+E_{p}\left(Z\right)I_{p}\frac{\partial^{2}\theta }{\partial Z^{2}}\right]\\ \; & -G_{p}\left(Z\right)A_{p}\kappa_{s}\left(\frac{\partial Y}{\partial Z}-\theta \right)=0 \end{array}$$

By substituting the following dimensionless variables

(8) $$\begin{array}{cc} y & =\frac{Y}{L},\;z=\frac{Z}{L},\;t=\sqrt{\frac{E_{0}I_{p}}{\left(\rho_{0}A_{p}+m_{f}\right)}}\;\frac{T}{L^{2}},\\ \beta & =\frac{m_{f}}{m_{f}+\rho_{0}A_{p}},\;u=\sqrt{\frac{m_{f}}{E_{0}I_{p}}}\;UL,\;\Lambda =\frac{G_{0}A_{p}\kappa_{s}L^{2}}{E_{0}I_{p}},\;\sigma =\frac{\rho_{0}I_{p}+\rho_{f}I_{f}}{L^{2}\left(\rho_{0}A_{p}+m_{f}\right)},\\ \alpha (z) & =\frac{E_{p}\left(z\right)}{E_{0}}=\frac{G_{p}\left(z\right)}{G_{0}},\;\gamma \left(z\right)=\frac{m_{f}+\rho \left(z\right)A_{p}}{m_{f}+\rho_{0}A_{p}},\;\delta \left(z\right)=\frac{\rho_{f}I_{f}+\rho \left(z\right)I_{p}}{\rho_{f}I_{f}+\rho_{0}I_{p}} \end{array}$$

where $E_{0}$, $\rho_{0}$ and $G_{0}$ denote the Young’s modulus, mass density and shear modulus at the left end of the pipe (z=0), respectively, with $G_{0}=E_{0}/\left[2\left(1+\nu \right)\right]$ for a homogeneous reference Poisson’s ratio ν. Under this definition, the dimensionless forms of Equations (6) and (7) become

(9) $$\begin{array}{cc} \; & \frac{\partial^{2}y}{\partial t^{2}}+2\sqrt{\beta }u\frac{1}{\gamma (z)}\frac{\partial^{2}y}{\partial z\partial t}+u^{2}\frac{1}{\gamma (z)}\frac{\partial^{2}y}{\partial z^{2}}\\ \; & -\Lambda \frac{\alpha (z)}{\gamma (z)}\frac{\partial^{2}y}{\partial z^{2}}+\Lambda \frac{\alpha (z)}{\gamma (z)}\frac{\partial \theta }{\partial z}-\Lambda \frac{\alpha^{\prime }\left(z\right)}{\gamma (z)}\frac{\partial y}{\partial z}+\Lambda \frac{\alpha^{\prime }\left(z\right)}{\gamma (z)}\theta =0 \end{array}$$

(10) $$\frac{\partial^{2}\theta }{\partial t^{2}}-\frac{1}{\sigma }\frac{\alpha^{\prime }\left(z\right)}{\delta (z)}\frac{\partial \theta }{\partial z}-\frac{1}{\sigma }\frac{\alpha (z)}{\delta (z)}\frac{\partial^{2}\theta }{\partial z^{2}}-\frac{\Lambda }{\sigma }\frac{\alpha (z)}{\delta (z)}\frac{\partial y}{\partial z}+\frac{\Lambda }{\sigma }\frac{\alpha (z)}{\delta (z)}\theta =0$$

The non-dimensional boundary constraints for both clamped ends are expressed as

(11) $$y\left(0,t\right)=0,\;y\left(1,t\right)=0,\;\frac{\partial y(0,t)}{\partial z}=0,\;\frac{\partial y(1,t)}{\partial z}=0,$$

The initial conditions of the system are expressed as follows:

(12) $$y\left(z,0\right)=0,\;\frac{\partial y(z,0)}{\partial t}=O\left(10^{-3}\right)\;\mathrm{random}\;\mathrm{noise}$$

in which $O(10^{-3})$ denotes a stochastic noise term.

## 3. Integral Transform Solution

### 3.1. Eigenfunction Expansion

The solution to the coupled governing Equations (9) and (10) is obtained via the analytic–numeric generalized integral transform technique (GITT) [39,40,47,48,49,50,51,52]. For a clamped–clamped Timoshenko pipe, Gu et al. [40] adopted the eigenfunction expansion below for the transverse displacement y(z,t); the present AFG extension retains the same spatial basis and solution procedure:

(13) $$\phi_{i}\left(z\right)=\left\{\begin{array}{c} \frac{\cos[\lambda_{i}\left(z-0.5\right)]}{\cos(\lambda_{i}/2)}-\frac{\cosh[\lambda_{i}\left(z-0.5\right)]}{\cosh(\lambda_{i}/2)}\;\mathrm{for}\;i\;\mathrm{odd},\\ \frac{\sin[\lambda_{i}\left(z-0.5\right)]}{\sin(\lambda_{i}/2)}-\frac{\sinh[\lambda_{i}\left(z-0.5\right)]}{\sinh(\lambda_{i}/2)}\;\mathrm{for}\;i\;\mathrm{even}, \end{array}\right.$$

where the corresponding eigenvalues $\lambda_{i}$ are obtained from the transcendental equations

(14) $$\tanh\left(\lambda_{i}/2\right)=\left\{\begin{array}{c} -\tan(\lambda_{i}/2)\;\mathrm{for}\;i\;\mathrm{odd},\\ \tan(\lambda_{i}/2)\;\mathrm{for}\;i\;\mathrm{even}, \end{array}\right.$$

For the rotational field, Gu et al. [40] take $\varphi_{i}\left(z\right)=\sin\left(i\pi z\right)$ with eigenvalues $\mu_{i}=i\pi$. The eigenfunctions $\phi_{i}\left(z\right)$ and $\varphi_{i}\left(z\right)$ satisfy the clamped–clamped boundary conditions and orthogonality relations $\int_{0}^{1}\phi_{i}\phi_{j}\;dz=\delta_{ij}M_{i}$ and $\int_{0}^{1}\varphi_{i}\varphi_{j}\;dz=\delta_{ij}N_{i}$, where $M_{i}=\int_{0}^{1}\phi_{i}^{2}\;dz$, $N_{i}=\int_{0}^{1}\varphi_{i}^{2}\;dz$, and the normalized forms are ${\overset{˜}{\phi }}_{i}=\phi_{i}/\sqrt{M_{i}}$, ${\overset{˜}{\varphi }}_{i}=\varphi_{i}/\sqrt{N_{i}}$.

Subsequently, the integral transformation pairs are established for the transformation and inversion processes:

(15) $$\left\{\begin{array}{cc} {\overset{¯}{y}}_{i}\left(t\right)=\int_{0}^{1}{\overset{˜}{\phi }}_{i}\left(z\right)y\left(z,t\right)dz,\; & \mathrm{transform}\\ y\left(z,t\right)=\sum_{i=1}^{\infty }{\overset{˜}{\phi }}_{i}\left(z\right){\overset{¯}{y}}_{i}\left(t\right),\; & \mathrm{inversion} \end{array}\right.$$

(16) $$\left\{\begin{array}{cc} {\overset{¯}{\theta }}_{i}\left(t\right)=\int_{0}^{1}{\overset{˜}{\varphi }}_{i}\left(z\right)\theta \left(z,t\right)dz,\; & \mathrm{transform}\\ \theta \left(z,t\right)=\sum_{i=1}^{\infty }{\overset{˜}{\varphi }}_{i}\left(z\right){\overset{¯}{\theta }}_{i}\left(t\right),\; & \mathrm{inversion} \end{array}\right.$$

### 3.2. Transformed ODE System

To perform the integral transformation of Equation (9), the equation is multiplied by the eigenfunction ${\overset{˜}{\phi }}_{i}\left(z\right)dz$ and integrated over the spatial domain *z* [0,1]. Then, using the inversion formula in Equation (15) leads to

(17) $$\begin{array}{cc} \; & \frac{d^{2}{\overset{¯}{y}}_{i}\left(t\right)}{dt^{2}}+2\sqrt{\beta }u\sum_{j=1}^{\infty }A_{ij}\frac{d{\overset{¯}{y}}_{j}\left(t\right)}{dt}+u^{2}\sum_{j=1}^{\infty }B_{ij}{\overset{¯}{y}}_{j}\left(t\right)\\ \; & \;-\Lambda \sum_{j=1}^{\infty }C_{ij}{\overset{¯}{y}}_{j}\left(t\right)+\Lambda \sum_{m=1}^{\infty }D_{im}{\overset{¯}{\theta }}_{m}\left(t\right)-\Lambda \sum_{j=1}^{\infty }E_{ij}{\overset{¯}{y}}_{j}\left(t\right)\\ \; & \;+\Lambda \sum_{m=1}^{\infty }F_{im}{\overset{¯}{\theta }}_{m}\left(t\right)=0,\;i=1,2,3,\ldots \end{array}$$

Similarly, by applying the integral operator $\int_{0}^{1}{\overset{˜}{\varphi }}_{m}\left(z\right)dz$ to Equation (10), the resulting ordinary differential equation is obtained as

(18) $$\begin{array}{c} \frac{d^{2}{\overset{¯}{\theta }}_{m}\left(t\right)}{dt^{2}}-\frac{1}{\sigma }\sum_{n=1}^{\infty }G_{mn}{\overset{¯}{\theta }}_{n}\left(t\right)-\frac{1}{\sigma }\sum_{n=1}^{\infty }H_{mn}{\overset{¯}{\theta }}_{n}\left(t\right)-\frac{\Lambda }{\sigma }\sum_{i=1}^{\infty }I_{mi}{\overset{¯}{y}}_{i}\left(t\right)\\ +\frac{\Lambda }{\sigma }\sum_{n=1}^{\infty }J_{mn}{\overset{¯}{\theta }}_{n}\left(t\right)=0,\;m=1,2,3,\ldots , \end{array}$$

where the coefficients contained in the ordinary differential system are defined by

(19) $$\left\{\begin{array}{cc} A_{ij}=\int_{0}^{1}\frac{1}{\gamma (z)}{\overset{˜}{\phi }}_{i}\left(z\right)\frac{d{\overset{˜}{\phi }}_{j}\left(z\right)}{dz}dz, & B_{ij}=\int_{0}^{1}\frac{1}{\gamma (z)}{\overset{˜}{\phi }}_{i}\left(z\right)\frac{d^{2}{\overset{˜}{\phi }}_{j}\left(z\right)}{dz^{2}}dz,\;\\ C_{ij}=\int_{0}^{1}\frac{\alpha (z)}{\gamma (z)}{\overset{˜}{\phi }}_{i}\left(z\right)\frac{d^{2}{\overset{˜}{\phi }}_{j}\left(z\right)}{dz^{2}}dz, & D_{im}=\int_{0}^{1}\frac{\alpha (z)}{\gamma (z)}{\overset{˜}{\phi }}_{i}\left(z\right)\frac{d{\overset{˜}{\varphi }}_{m}\left(z\right)}{dz}dz,\;\\ E_{ij}=\int_{0}^{1}\frac{\alpha^{\prime }\left(z\right)}{\gamma (z)}{\overset{˜}{\phi }}_{i}\left(z\right)\frac{d{\overset{˜}{\phi }}_{j}\left(z\right)}{dz}dz, & F_{im}=\int_{0}^{1}\frac{\alpha^{\prime }\left(z\right)}{\gamma (z)}{\overset{˜}{\phi }}_{i}\left(z\right){\overset{˜}{\varphi }}_{m}\left(z\right)dz,\;\\ G_{mn}=\int_{0}^{1}\frac{\alpha^{\prime }\left(z\right)}{\delta (z)}{\overset{˜}{\varphi }}_{m}\left(z\right)\frac{d{\overset{˜}{\varphi }}_{n}\left(z\right)}{dz}dz, & H_{mn}=\int_{0}^{1}\frac{\alpha (z)}{\delta (z)}{\overset{˜}{\varphi }}_{m}\left(z\right)\frac{d^{2}{\overset{˜}{\varphi }}_{n}\left(z\right)}{dz^{2}}dz,\;\\ I_{mi}=\int_{0}^{1}\frac{\alpha (z)}{\delta (z)}{\overset{˜}{\varphi }}_{m}\left(z\right)\frac{d{\overset{˜}{\phi }}_{i}\left(z\right)}{dz}dz, & J_{mn}=\int_{0}^{1}\frac{\alpha (z)}{\delta (z)}{\overset{˜}{\varphi }}_{m}\left(z\right){\overset{˜}{\varphi }}_{n}\left(z\right)dz, \end{array}\right.$$

### 3.3. Time Integration and Response Extraction

The eigenfunctions automatically satisfy the boundary constraints. Transforming the initial conditions in Equation (12) gives ${\overset{¯}{y}}_{i}\left(0\right)=0$ and ${\dot{\overset{¯}{y}}}_{i}\left(0\right)=\int_{0}^{1}{\overset{˜}{\phi }}_{i}\left(z\right)\;\partial y\left(z,0\right)/\partial t\;dz$. For the determination of ${\overset{¯}{y}}_{i}\left(t\right)$ and ${\overset{¯}{\theta }}_{m}\left(t\right)$, the infinite set of ordinary linear differential equations given by Equations (17) and (18) with these initial conditions is reduced to a finite truncation order *N*, thereby satisfying the required accuracy. The derived governing system is solved by means of the DIVPAG subroutine in the IMSL Library [53], which possesses an automatic error control strategy. In this study, a convergence criterion of $10^{-6}$ is adopted. Finally, the dimensionless function $\;y\left(z,t\right)$ is recovered by the analytical inversion formula in Equation (15) once the numerical solution of ${\overset{¯}{y}}_{i}\left(t\right)$ is determined. From the stabilized segment of $y(z_{m},t)$ at mid-span ($z_{m}=0.5$), the first two spectral peaks yield $\omega_{1}$ and $\omega_{2}$, and the maximum deflection $y_{M}=\max_{t}\left|y\left(z_{m},t\right)\right|$ is recorded for each parameter triplet.

## 4. MLP Surrogate Model

Although the GITT formulation presented in Section 3 provides high-fidelity solutions, repeated numerical simulations over the multi-dimensional parameter space (k,u,L/D) remain expensive because each case requires a complete eigendecomposition, spatial integration, series truncation, and time-domain solution. This cost limits the number of parametric cases that can be explored with online GITT alone.

To overcome this limitation, a data-driven surrogate model based on a multi-layer perceptron (MLP) feedforward neural network is developed in this section. The MLP learns the input–output mapping from a GITT-generated dataset and replaces repeated online numerical solutions with fast forward inference. The governing equations and the GITT solution remain the physical and numerical benchmark; the MLP only approximates the discrete mapping between design parameters and target responses. Table 1 summarizes the complementary roles of the two approaches adopted in this study.

### 4.1. Problem Formulation

Let the input vector be defined as

(20) $$x={\left[u,\;k,\;L/D\right]}^{T}\in R^{3},$$

where *u* is the dimensionless flow velocity, *k* is the power-law index controlling the axial material gradation, and L/D is the aspect ratio of the pipe.

The output vector of interest is

(21) $$y={\left[\omega_{1},\;\omega_{2},\;y_{M}\right]}^{T}\in R^{3},$$

where $\omega_{1}$ and $\omega_{2}$ denote the first- and second-mode dimensionless natural angular frequencies, and $y_{M}$ is the dimensionless maximum transverse deflection.

The objective is to construct three surrogate mappings

(22) y^i=fi(x;Θi),i=1,2,3,

such that y^1≈ω1, y^2≈ω2, and y^3≈yM, where $\Theta_{i}$ denotes the trainable parameters of the *i*th network.

### 4.2. Training Dataset Generated by GITT

A labeled dataset

(23) $$D={\left\{(x^{\left(n\right)},\;y^{\left(n\right)})\right\}}_{n=1}^{N_{s}},$$

is generated offline by the GITT solver described in Section 3. For each sample *n*, the input parameters $\left(u^{\left(n\right)},k^{\left(n\right)},L/D^{\left(n\right)}\right)$ are prescribed, and the corresponding GITT outputs $\left(\omega_{1}^{\left(n\right)},\omega_{2}^{\left(n\right)},y_{M}^{\left(n\right)}\right)$ are recorded. In the present study, $N_{s}=336$ samples are used. The parameter ranges follow those listed in Table 2. The GITT dataset serves as the ground truth for surrogate training and independent testing.

### 4.3. Interior Block Train–Test Partition

To assess generalization rather than in-sample interpolation, the full dataset D is partitioned into a training set

(24) $$D_{\mathrm{tr}}={\left\{(x^{\left(n\right)},\;y^{\left(n\right)})\right\}}_{n=1}^{N_{\mathrm{tr}}},$$

and an independent test set

(25) $$D_{\mathrm{te}}={\left\{(x^{\left(n\right)},\;y^{\left(n\right)})\right\}}_{n=1}^{N_{\mathrm{te}}},$$

with $N_{\mathrm{tr}}+N_{\mathrm{te}}=N_{s}$. A fixed 80/20 split is adopted, yielding $N_{\mathrm{tr}}=270$ training samples and $N_{\mathrm{te}}=66$ test samples. Because the $N_{s}=336$ GITT samples are organized as ordered parameter sweeps—*u*-sweeps at fixed (k,L/D) and L/D-sweeps at fixed (u,k)—the dataset is first divided into 16 contiguous blocks. Within each block, only interior samples of the varying parameter are eligible for testing: for *u*-sweeps, endpoints u=0 and $u=u_{\max}$ are excluded; for L/D-sweeps at u=3, endpoints L/D=5 and L/D=99 are excluded. Test picks are then allocated proportionally to block size and selected by even spacing inside each block; all remaining samples form $D_{\mathrm{tr}}$. Samples in $D_{\mathrm{te}}$ are strictly excluded from network training, min–max normalization, and all test-set accuracy metrics reported in Table 3 and Section 5.2.1; training-set reference metrics are listed separately in Table 4.

### 4.4. Data Preprocessing

Because the input variables and output responses have different magnitudes, min–max normalization is applied to improve training stability and convergence. For each input component $x_{j}$ (j=1,2,3), the normalized variable is

(26) $${\overset{˜}{x}}_{j}=\frac{x_{j}-x_{j,\min}}{x_{j,\max}-x_{j,\min}},$$

where $x_{j,\min}$ and $x_{j,\max}$ are the minimum and maximum values of the *j*th input over the *training* set $D_{\mathrm{tr}}$, so that ${\overset{˜}{x}}_{j}\in \left[0,1\right]$.

For the outputs, because $y_{M}$ is several orders of magnitude smaller than $\omega_{1}$ and $\omega_{2}$, a logarithmic transform is applied before normalization to reduce relative-error bias:

(27) $${\overset{ˇ}{y}}_{3}=\ln\left(y_{M}\right).$$

The transformed output vector is defined as

(28) $$\overset{ˇ}{y}={\left[\omega_{1},\;\omega_{2},\;{\overset{ˇ}{y}}_{3}\right]}^{T},$$

and each component ${\overset{ˇ}{y}}_{i}$ is normalized by

(29) $${\overset{˜}{y}}_{i}=\frac{{\overset{ˇ}{y}}_{i}-{\overset{ˇ}{y}}_{i,\min}}{{\overset{ˇ}{y}}_{i,\max}-{\overset{ˇ}{y}}_{i,\min}},\;i=1,2,3.$$

The normalization parameters for inputs and outputs are stored and reused during inference.

### 4.5. Network Architecture

Instead of a single network with three outputs, three independent MLPs are trained, one for each response variable. This decoupled strategy prevents scale differences among $\omega_{1}$, $\omega_{2}$, and $y_{M}$ from biasing the shared loss function and allows for output-specific hyperparameter tuning.

Each surrogate model $f_{i}\left(x;\Theta_{i}\right)$ is a fully connected feedforward network with the following structure:

- Input layer: three neurons corresponding to $\overset{˜}{x}={\left[\overset{˜}{u},\overset{˜}{k},\overset{˜}{L/D}\right]}^{T}$;
- First hidden layer: 45 neurons with hyperbolic tangent activation;
- Second hidden layer: 25 neurons with hyperbolic tangent activation;
- Output layer: one neuron with linear activation.

Let $z^{\left(0\right)}=\overset{˜}{x}$. The forward propagation for hidden layer ℓ=1,2 is

(30) $$z^{\left(\ell \right)}=\sigma \;\left(W^{\left(\ell \right)}z^{\left(\ell -1\right)}+b^{\left(\ell \right)}\right),$$

where $W^{\left(\ell \right)}$ and $b^{\left(\ell \right)}$ are the weight matrix and bias vector of layer *ℓ*, and σ(·) denotes the element-wise hyperbolic tangent activation:

(31) $$\sigma \left(z\right)=\tanh\left(z\right)=\frac{e^{z}-e^{-z}}{e^{z}+e^{-z}}.$$

The output layer uses a linear activation:

(32) $${\overset{˜}{y}}_{i}=w^{(3)T}z^{\left(2\right)}+b^{\left(3\right)}.$$

Collecting all weights and biases into $\Theta_{i}$, the surrogate model can be compactly written as

(33) $${\overset{˜}{y}}_{i}=f_{i}\left(\overset{˜}{x};\Theta_{i}\right),\;i=1,2,3.$$

According to the universal approximation theorem, a feedforward network with at least one hidden layer and sufficient neurons can approximate any continuous multivariate function on a compact domain to arbitrary accuracy. This theoretical property supports the use of MLPs as surrogate models for smooth parametric mappings extracted from GITT solutions.

The tanh activation function was selected for its smoothness and zero-centered output range (−1,1), which suit the L-BFGS quasi-Newton optimizer and facilitate the accurate representation of the smooth GITT response surfaces. While a systematic grid search over alternative activations (e.g., ReLU, logistic sigmoid) and network architectures was beyond the scope of the present surrogate study, the chosen [45,25]–tanh configuration proved sufficient to achieve $R^{2}>0.99$ on the independent test set; a more exhaustive architecture optimization can be pursued in future work.

### 4.6. Loss Function and Training Algorithm

For the *i*th output, given the normalized training pairs ${\left\{\left({\overset{˜}{x}}^{\left(n\right)},{\overset{˜}{y}}_{i}^{\left(n\right)}\right)\right\}}_{n=1}^{N_{\mathrm{tr}}}$, the mean squared error (MSE) loss is minimized:

(34) $$L_{i}\left(\Theta_{i}\right)=\frac{1}{N_{\mathrm{tr}}}\sum_{n=1}^{N_{\mathrm{tr}}}{\left[{\overset{˜}{y}}_{i}^{\left(n\right)}-f_{i}\left({\overset{˜}{x}}^{\left(n\right)};\Theta_{i}\right)\right]}^{2}.$$

The network parameters are obtained by

(35) $$\Theta_{i}^{⋆}=\arg\min_{\Theta_{i}}L_{i}\left(\Theta_{i}\right),\;i=1,2,3.$$

Training is performed with the limited-memory Broyden–Fletcher–Goldfarb–Shanno (L-BFGS) quasi-Newton algorithm (lbfgs in scikit-learn), which provides stable convergence for the present small regression networks. Only samples in $D_{\mathrm{tr}}$ ($N_{\mathrm{tr}}=270$) participate in training; samples in $D_{\mathrm{te}}$ ($N_{\mathrm{te}}=66$) are reserved for independent accuracy assessment in Section 5.2.1. For $\omega_{1}$ only, sample weights are applied to emphasize the pre-critical regime (u≥4 at L/D=50), samples with large $y_{M}$, and the intermediate power-law index k=0.5, where the GITT curve exhibits the sharpest local variation. The maximum number of iterations is set to 3000 for $\omega_{1}$ and $\omega_{2}$, and 5000 for $y_{M}$. On the authors’ platform, the full offline training of the three networks completes in a few seconds. After training, the network parameters and normalization constants are saved for subsequent fast inference.

### 4.7. Inverse Transformation and Inference

For a new query input $x^{⋆}$, the prediction procedure consists of four steps. First, the input is normalized using Equation (26):

(36) $${\overset{˜}{x}}^{⋆}={\mathrm{Norm}}_{x}\left(x^{⋆}\right).$$

Second, forward propagation is performed through the three trained networks:

(37) $${\overset{˜}{y}}_{i}^{⋆}=f_{i}\left({\overset{˜}{x}}^{⋆};\Theta_{i}^{⋆}\right),\;i=1,2,3.$$

Third, the outputs are denormalized using the inverse of Equation (29):

(38) $${\overset{ˇ}{y}}_{i}^{⋆}={\overset{˜}{y}}_{i}^{⋆}\;\left({\overset{ˇ}{y}}_{i,\max}-{\overset{ˇ}{y}}_{i,\min}\right)+{\overset{ˇ}{y}}_{i,\min},\;i=1,2,3.$$

Finally, the physical responses are recovered as

(39) ω^1=yˇ1⋆,ω^2=yˇ2⋆,y^M=expyˇ3⋆,

and the predicted vector is y^⋆=[ω^1,ω^2,y^M]T. Once trained, the surrogate model reduces each new prediction to a few matrix–vector multiplications and activation evaluations, thereby avoiding repeated GITT simulations. The accuracy metrics and computational efficiency of the trained model are quantified in Section 5.2.

### 4.8. Remarks on Surrogate Validity

The MLP surrogate is a metamodel fitted to GITT-generated samples within the parameter ranges u∈[0,6.2], k∈[0,10], and L/D∈[5,99]. The following limitations should be noted when interpreting surrogate predictions. (i) Extrapolation outside the training domain—particularly to flow velocities above the highest critical velocity in the dataset, to power-law indices k>10, or to aspect ratios beyond [5,99]—may yield unreliable results and should be avoided unless supplemented with additional GITT verification samples. (ii) The surrogate predicts scalar outputs (modal frequencies and maximum deflection), but does not replace time-history or spectral post-processing obtained directly from GITT (Section 5.1.1). (iii) If the training dataset is updated, the material system is changed, or the parameter ranges are extended, the MLP should be retrained and the saved normalization parameters and network weights refreshed accordingly. (iv) The present surrogate is specific to the Alumina–Steel material pair and clamped–clamped boundary conditions; extending it to other material combinations or boundary conditions requires generating a new GITT dataset and retraining. These limitations do not reduce the value of the surrogate for rapid parametric evaluation, design exploration, and sensitivity analysis within the validated domain. Table 5 summarizes the MLP surrogate model formulation.

## 5. Results and Discussion

This section reports the vibration characteristics of the Alumina–Steel AFG pipe system summarized in Table 2. The numerical simulations were implemented in Fortran using the GITT procedure to investigate how the power-law gradation index *k*, dimensionless flow velocity *u*, and aspect ratio L/D influence the modal frequencies and maximum deflection of fluid-conveying pipes modeled as Timoshenko beams. Gu et al. [40] applied the same GITT procedure to study the effect of aspect ratio on a homogeneous Timoshenko pipe and demonstrated convergence by adjusting the modal truncation order; Gu et al. [39] reported similar convergence for flow-velocity sweeps. For the present Alumina–Steel AFG configuration, the spatial variation of the dimensionless coefficient functions α(z), γ(z), and δ(z) introduced by the power-law gradation remains smooth (on z∈[0,1] for finite *k*); consequently, the eigenfunction expansion retains the same exponential convergence rate established in Refs. [39,40]. Setting N=20 therefore maintains the relative truncation error well below the $10^{-6}$ tolerance adopted throughout this work. Based on these prior results, a truncation order of N=20 is adopted for all calculations.

For the subsequent calculations, an Alumina–Steel Functionally Graded (AFG) pipe is considered. The material properties vary along the pipe length according to a power-law distribution, transitioning from pure alumina (Al_2_O_3_, *E* = 390 GPa, ρ = 3960 kg/m^3^) at the left end to steel (*E* = 210 GPa, ρ = 7800 kg/m^3^) at the right end. Table 2 summarizes the primary simulation parameters. In the present study, the aspect ratio varies between 5 and 100. The dimensionless flow velocity is scanned from 0 to 6.2 in the GITT dataset; the value 2π marks the critical-velocity scale for the reference alumina-rich case (k=0).

### 5.1. GITT Parametric Results

This subsection presents the high-fidelity GITT solutions for the vibration characteristics of the Alumina–Steel AFG pipe defined in Table 2. The results establish the physics-based benchmark used later for MLP surrogate validation in Section 5.2.

#### 5.1.1. Numerical Setup and Time-Domain Response

**Figure 3:**
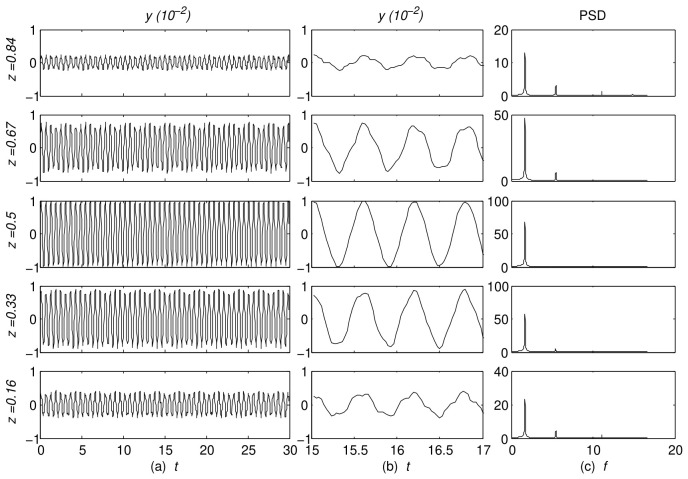
Simulated dynamic response for k=10.0, u=3.0, L/D=50. (**a**): Time history of dimensionless transverse deflection at five equally spaced locations along the pipe over the entire simulation. (**b**): Same deflection during the reduced time window t∈ [15,17]; (**c**): Corresponding frequency spectrum.

Figure 3 depicts the typical time histories and response frequencies for the case of k=10.0, u=3.0, L/D=50. The first column presents the dimensionless displacement *y* at five equidistant spatial points along the pipe, while the second column zooms in on the time interval t∈ [15, 17]. The third column displays the corresponding frequency spectrum. As indicated by the long-term stability in the first column and the steady oscillations in the second, the system maintains a stable vibration. The spectral analysis reveals multiple frequency peaks, suggesting that the dynamic response involves multi-mode contributions.

#### 5.1.2. Effect of Flow Velocity and Power-Law Index

**Figure 4:**
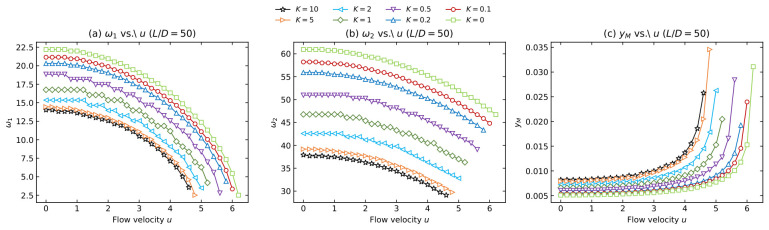
GITT parametric results at L/D=50 as functions of the dimensionless flow velocity *u* for power-law indices k=0–10: (**a**) first-mode natural frequency $\omega_{1}$; (**b**) second-mode natural frequency $\omega_{2}$; (**c**) maximum vibration deflection $y_{M}$.

The dimensionless natural angular frequencies and maximum deflection as functions of the flow velocity *u* and the power-law index *k* (at the eight discrete values k∈{0,0.1,0.2,0.5,1,2,5,10} sampled in the GITT dataset) at L/D=50 are presented in Figure 4. The pure-steel limit k→∞ is discussed as a qualitative asymptotic reference and is not included in the GITT dataset or the plotted curves.

Panel (a) of Figure 4 demonstrates that the fundamental frequency $\omega_{1}$ decreases as *u* increases. Notably, for k=0 (pure alumina), the critical dimensionless velocity approaches 2π, a finding consistent with the results of Gu et al. [39]. Furthermore, at a constant flow velocity, the frequency diminishes as the power-law index *k* rises. Consequently, the critical flow velocity also decreases with increasing *k*. The second-mode frequencies $\omega_{2}$ in panel (b) exhibit a similar trend. Panel (c) shows the maximum vibration deflection as a function of the internal flow velocity for a range of power-law indices. A monotonic increase in deflection is observed as the flow velocity rises, a trend consistent with the findings of Guo and Lou [54] and Gu et al. [39]. As the velocity approaches the critical value, the deflection diverges towards infinity, signaling the onset of instability. Furthermore, higher power-law indices (e.g., k=10) result in significantly larger deflections compared to lower indices (e.g., k=5 to 0). The rate of increase in maximum deflection $y_{M}$ accelerates with larger values of *k*, indicating that pipes characterized by a higher power-law index are more susceptible to instability at a given flow velocity.

#### 5.1.3. Effect of Aspect Ratio

**Figure 5:**
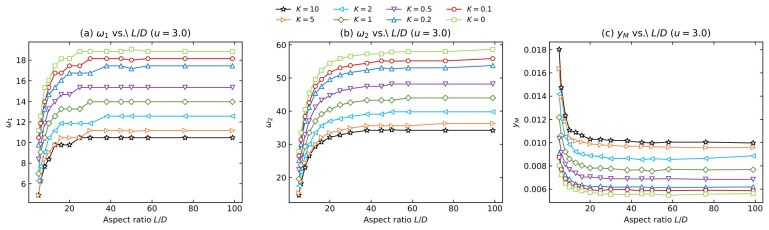
GITT parametric results at u=3.0 as functions of the aspect ratio L/D for power-law indices k=0–10: (**a**) first-mode natural frequency $\omega_{1}$; (**b**) second-mode natural frequency $\omega_{2}$; (**c**) maximum vibration deflection $y_{M}$.

Figure 5 presents the dimensionless natural angular frequencies and maximum deflection as functions of the aspect ratio L/D for the same eight discrete power-law indices k∈{0,0.1,0.2,0.5,1,2,5,10} at u=3.0.

It is observed that for smaller power-law indices, both $\omega_{1}$ and $\omega_{2}$ in panels (a) and (b) exhibit a more pronounced decrease as the aspect ratio diminishes. Notably, the frequencies converge to constant values when L/D exceeds 40 for $\omega_{1}$ and 60 for $\omega_{2}$. This trend is consistent with the Timoshenko-to-Euler transition reported by Gu et al. [40] for homogeneous pipes: at large L/D, shear effects weaken and the response approaches the slender-beam limit, whereas smaller aspect ratios require the full Timoshenko model. Additionally, the influence of the aspect ratio on the dynamic properties of the pipe is diminished as the power-law index increases. For any given aspect ratio, an increase in the power-law index yields a decrease in the frequencies, thereby underscoring the significant impact of material composition on the stability of AFG pipes.

Panel (c) of Figure 5 illustrates how the maximum vibration deflection varies with the aspect ratio. Here, the deflection at the pipe’s center is plotted for L/D ranging from 5 to 100. The results indicate that the maximum deflection initially decreases until a threshold aspect ratio is reached, beyond which it stabilizes at a constant value. Moreover, an inverse relationship is observed between the power-law index *k* and the maximum deflection; as *k* increases, the deflection diminishes. This implies that the vibration amplitude is maximized for a pure alumina pipe (k=0) and would approach its minimum in the asymptotic limit of a pure steel pipe (k→∞, treated here as a qualitative reference).

### 5.2. Validation of the MLP Surrogate Model

Following the GITT parametric study in Section 5.1, the MLP surrogate described in Section 4 is trained on $D_{\mathrm{tr}}$ ($N_{\mathrm{tr}}=270$) and evaluated on the independent test set $D_{\mathrm{te}}$ ($N_{\mathrm{te}}=66$). Together with Table 1, the results below demonstrate that the GITT–MLP surrogate workflow preserves the high-fidelity vibration characteristics revealed by GITT while enabling rapid online evaluation.

#### 5.2.1. Accuracy Assessment on Training and Test Sets

The surrogate accuracy is evaluated on both $D_{\mathrm{tr}}$ ($N_{\mathrm{tr}}=270$) and the independent test set $D_{\mathrm{te}}$ ($N_{\mathrm{te}}=66$) using the coefficient of determination

(40) Ri2=1−∑n=1Nyi(n)−y^i(n)2∑n=1Nyi(n)−yi,mean2,

the mean absolute error

(41) MAEi=1N∑n=1Nyi(n)−y^i(n),

the root mean square error

(42) RMSEi=1N∑n=1Nyi(n)−y^i(n)2,

the maximum relative error

(43) εi,max=max1≤n≤Nyi(n)−y^i(n)yi(n)×100%,

and the mean relative error

(44) εi,mean=1N∑n=1Nyi(n)−y^i(n)yi(n)×100%,

where $N=N_{\mathrm{tr}}$ for the training set and $N=N_{\mathrm{te}}$ for the test set, and $y_{i,\mathrm{mean}}=\frac{1}{N}\sum_{n=1}^{N}y_{i}^{\left(n\right)}$ is the sample mean of the *i*th GITT output.

Table 4 reports the in-sample reference accuracy on $D_{\mathrm{tr}}$. Because the training set retains parameter endpoints excluded from testing, the largest local errors appear near u=0, $u=u_{\max}$, and extreme L/D values.

Table 3 summarizes the independent test-set accuracy for $\omega_{1}$, $\omega_{2}$, and $y_{M}$. These 66 samples never participated in training or normalization.

**Figure 6:**
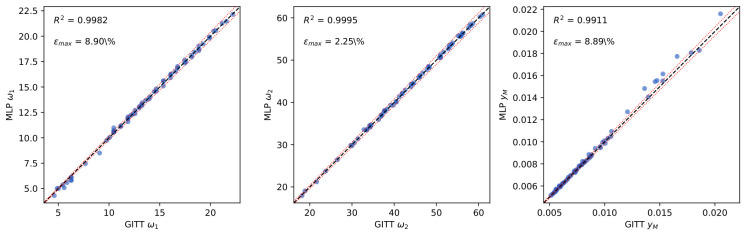
Parity plots comparing GITT reference values with MLP predictions for $\omega_{1}$, $\omega_{2}$, and $y_{M}$ on the independent test set ($N_{\mathrm{te}}=66$). The dashed line denotes y=x; dotted lines indicate ±2% bands.

Figure 6 presents parity plots comparing GITT reference values with MLP predictions for the three outputs on $D_{\mathrm{te}}$. The data points cluster closely around the diagonal line y=x, confirming good agreement on unseen samples. On the independent test set, $\omega_{1}$, $\omega_{2}$, and $y_{M}$ achieve $R^{2}=0.9982$, 0.9995, and 0.9911, with $\varepsilon_{\max}=8.90\%$, 2.25%, and 8.89%, and $\varepsilon_{\mathrm{mean}}=1.50\%$, 0.49%, and 1.45%, respectively.

#### 5.2.2. Physical Consistency with GITT Trends

Pointwise metrics in Table 3 and Table 4 quantify overall surrogate accuracy, but they do not by themselves guarantee that the MLP preserves the *physical trends* revealed by GITT. Therefore, representative parametric curves are reconstructed with the trained MLP and overlaid on the GITT reference data shown in Figure 4 and Figure 5.

For each sweep, the MLP is queried at the same input combinations (u,k,L/D) used to generate the GITT curves. The maximum relative error along each reconstructed curve is defined as

(45) εoverlay,max=maxn∈Sy(n)−y^(n)y(n)×100%,

where S denotes the set of GITT samples belonging to the selected sweep. For $\omega_{1}$ and $\omega_{2}$ vs. *u* with L/D=50, S contains all 224 samples with L/D=50 and k∈{0,0.1,…,10}; for $y_{M}$ vs. L/D with u=3.0, S contains all 120 samples with u=3.0 and varying *k* and L/D.

**Figure 7:**
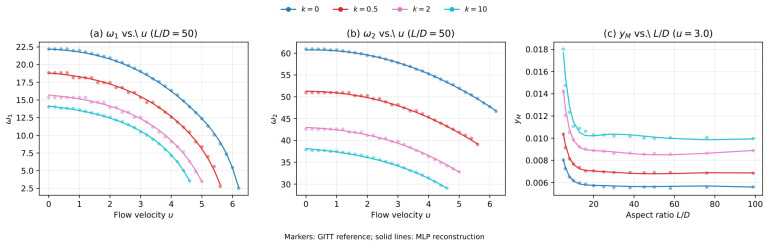
Overlay comparison between GITT reference curves (markers) and MLP reconstructions (solid lines) for representative parametric sweeps: (**a**) $\omega_{1}$ vs. *u* at L/D=50; (**b**) $\omega_{2}$ vs. *u* at L/D=50; (**c**) $y_{M}$ vs. L/D at u=3.0. Representative power-law indices k=0, 0.5, 2, and 10 are shown.

Figure 7 compares GITT markers with MLP solid-line reconstructions for three representative parametric sweeps: $\omega_{1}\left(u\right)$ and $\omega_{2}\left(u\right)$ at L/D=50 (panels a–b), and $y_{M}\left(L/D\right)$ at u=3.0 (panel c). Curves for k=0, 0.5, 2, and 10 are shown.

The MLP reproduces the same monotonic decrease of $\omega_{1}$ and $\omega_{2}$ with increasing flow velocity, the rapid growth of $y_{M}$ near the critical velocity, and the aspect-ratio-induced plateau of $y_{M}$ at large L/D. The maximum overlay error along any reconstructed curve is below 9%. Table 6 summarizes $\varepsilon_{\mathrm{overlay},\max}$ for the four sweeps corresponding to panels (a)–(c) of Figure 4 and Figure 5. All values remain below 9%, confirming that the surrogate preserves the GITT parametric trends within the sampled domain.

#### 5.2.3. Computational Efficiency and Practical Advantage

Let $T_{\mathrm{GITT}}$ denote the average wall-clock time required for one complete GITT analysis at a single parameter point and $T_{\mathrm{MLP}}$ denote the average time for one MLP inference. In the present study, the high-fidelity benchmark is implemented in Fortran: for each prescribed triplet (u,k,L/D), an independent executable run is launched, a raw output file is written, and post-processing extracts $\omega_{1}$, $\omega_{2}$, and $y_{M}$. No batching or reuse of intermediate eigenfunction data occurs between parameter points, so the cost scales linearly with the number of queries. The speedup factor is defined as

(46) $$\eta =\frac{T_{\mathrm{GITT}}}{T_{\mathrm{MLP}}}.$$

For a parametric sweep involving $N_{q}$ query points, the total computational costs are

(47) $$T_{\mathrm{total}}^{\mathrm{GITT}}=N_{q}\;T_{\mathrm{GITT}},\;T_{\mathrm{total}}^{\mathrm{MLP}}=T_{\mathrm{train}}+N_{q}\;T_{\mathrm{MLP}},$$

where $T_{\mathrm{train}}$ is the one-time offline MLP training cost. For the Fortran GITT benchmark, $T_{\mathrm{GITT}}$ at a fixed (k,L/D) was estimated from the wall-clock interval between consecutive raw output files generated during a full *u*-sweep (u=0–6.0, step 0.2, 30–31 points per sweep). Table 7 lists the results for k=10 and five representative aspect ratios. The per-query time increases from about 10 s at L/D=5 to about 122 s at L/D=50, reflecting the growing cost of the time-domain integration and series-truncated modal resolution as the pipe slenderness increases; completing one *u*-sweep therefore requires from 5 min (L/D=5) to 63 min (L/D=50). The entry $T_{\mathrm{GITT}}=47$ s used in Table 8 is the arithmetic mean over these five sweeps and serves as a representative single-query cost in the following comparison with the MLP.

Table 8 compares the mean GITT cost in Table 7 with MLP inference on the authors’ Python 3.10 workstation. MLP timings were obtained with benchmark_mlp_timing.py: $T_{\mathrm{train}}$ is the mean of three training runs (Section 4.6), and $T_{\mathrm{MLP}}$ is the average over $10^{4}$ sequential single-query forward passes. Because the trained MLP can be saved and reloaded, $T_{\mathrm{train}}$ is amortized over large sweeps and $N_{q}T_{\mathrm{MLP}}$ remains negligible compared with repeated Fortran GITT launches once $T_{\mathrm{GITT}}\gg T_{\mathrm{MLP}}$.

The breakeven number of queries, $N_{q}^{⋆}=T_{\mathrm{train}}/\left(T_{\mathrm{GITT}}-T_{\mathrm{MLP}}\right)$, is approximately 1; even a single post-training inference is already far cheaper than launching another Fortran GITT run. For design optimization, Monte Carlo simulation, or global sensitivity analysis requiring $O(10^{4})$–$O(10^{5})$ evaluations (Section 6), repeated GITT runs would require about 131 h of wall-clock time for $N_{q}=10^{4}$ at the mean $T_{\mathrm{GITT}}=47$ s in Table 7 (about 28 h at L/D=5, about 339 h at L/D=50), whereas the trained MLP completes the same $10^{4}$ evaluations in about 1.6 s after one-time offline training (≈9 s for $N_{q}=10^{5}$). This practical advantage validates the complementary roles summarized in Table 1: GITT provides the physics-based benchmark, while the MLP enables downstream tasks that would otherwise be computationally prohibitive.

## 6. Global Sensitivity Analysis

After validating the MLP surrogate in Section 5.2, it is used as an inexpensive evaluator for global sensitivity analysis (GSA) [55]. The objective is to quantify how the power-law gradation index *k*, dimensionless flow velocity *u*, and aspect ratio L/D govern the vibration characteristics ($\omega_{1}$, $\omega_{2}$, and $y_{M}$) of the Alumina–Steel AFG pipe. Three complementary methods are employed: variance-based Sobol indices, SHAP values [56], and partial dependence plots (PDPs).

### 6.1. Sobol Sensitivity Indices

**Figure 8:**
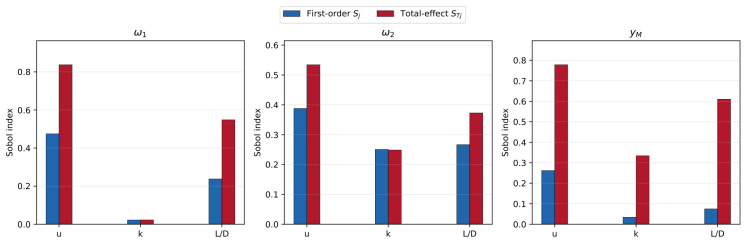
First-order ($S_{j}$, blue) and total-effect ($S_{Tj}$, red) Sobol sensitivity indices for each input *u*, *k*, and L/D on $\omega_{1}$, $\omega_{2}$, and $y_{M}$. For example, the red bar above *u* in the $\omega_{1}$ panel is $S_{T,u}$.

For input $x_{j}$ (j=1,2,3 corresponding to *u*, *k*, and L/D), the first-order Sobol index is defined as

(48) $$S_{j}=\frac{{\mathrm{Var}}_{x_{j}}\;\left[E_{x_{∼j}}\left(Y|x_{j}\right)\right]}{\mathrm{Var}(Y)},$$

where *Y* denotes a scalar output ($\omega_{1}$, $\omega_{2}$, or $y_{M}$), $x_{∼j}$ represents all inputs except $x_{j}$, and Var(·) denotes the variance operator. The total-effect Sobol index is

(49) $$S_{Tj}=1-\frac{{\mathrm{Var}}_{x_{∼j}}\;\left[E_{x_{j}}\left(Y|x_{∼j}\right)\right]}{\mathrm{Var}(Y)},$$

which includes both the direct effect of $x_{j}$ and its interactions with other inputs. The indices are estimated by Saltelli sampling [55] with N=512 base points (about N(2D+2)=4096 MLP evaluations per output, D=3) over the parameter ranges covered by the GITT dataset (u∈[0,6.2], k∈[0,10], L/D∈[5,99]), using the trained MLP as the response evaluator. Because each MLP evaluation requires only forward propagation, such sample sizes are affordable. The estimated first-order and total-effect indices are summarized in Figure 8.

As shown in Figure 8, the red bars give the total-effect indices $S_{T,u}$, $S_{T,k}$, and $S_{T,L/D}$ for the corresponding abscissa labels; the blue bars give the first-order indices $S_{u}$, $S_{k}$, and $S_{L/D}$. For $\omega_{1}$, *u* is the dominant factor ($S_{T,u}\approx 0.84$), followed by L/D ($S_{T,L/D}\approx 0.55$); the contribution of *k* is negligible ($S_{T,k}\approx 0.02$). For $\omega_{2}$, *u*, *k*, and L/D all contribute noticeably ($S_{T,u}\approx 0.53$, $S_{T,k}\approx 0.25$, $S_{T,L/D}\approx 0.37$). For $y_{M}$, *u* and L/D exhibit the largest total effects ($S_{T,u}\approx 0.78$, $S_{T,L/D}\approx 0.61$), with a secondary contribution from *k* ($S_{T,k}\approx 0.33$), indicating strong interaction among the inputs near the critical flow regime.

### 6.2. SHAP Feature Importance

SHAP (SHapley Additive exPlanations) values provide a model-agnostic measure of feature contribution for each prediction. For input feature *j* and output *Y*, the SHAP value $\phi_{j}$ satisfies the additive explanation

(50) Y^=ϕ0+∑j=13ϕj,

where $\phi_{0}$ is the baseline prediction, approximated as the mean MLP output over the background set. The Shapley value is defined as

(51) $$\phi_{j}=\sum_{S\subseteq \{1,2,3\}\setminus \{j\}}\frac{|S|!\;(3-|S|-1)!}{3!}\left[f_{S\cup \{j\}}\left(x_{S\cup \{j\}}\right)-f_{S}\left(x_{S}\right)\right],$$

where $f_{S}\left(x_{S}\right)=E\left[f\left(x\right)|x_{S}\right]$ denotes the expected MLP response when the features in S are fixed and the remaining inputs are marginalized over the training domain. KernelSHAP approximates these conditional expectations using a background set of $N_{\mathrm{bg}}=120$ inputs resampled from the GITT dataset, with 100 coalition samples per explained instance. The mean absolute SHAP value,

(52) $$I_{j}=E\left[|\phi_{j}|\right],$$

is used to rank the global importance of each input for a given output; in practice, $I_{j}$ is computed as the sample mean of $|\phi_{j}|$ over the background set. Table 9 lists the results; because the three outputs have different scales, the relative ranking within each row is emphasized. For $\omega_{1}$, *u* is the most important feature ($I_{u}\approx 5.06$), whereas *k* and L/D have comparable magnitudes ($I_{k}\approx 2.85$, $I_{L/D}\approx 2.75$), consistent with the Sobol ordering in Figure 8. For $\omega_{2}$, SHAP ranks *k* first ($I_{k}\approx 6.94$), followed by L/D ($I_{L/D}\approx 3.43$) and *u* ($I_{u}\approx 2.56$), whereas the Sobol total-effect indices place *u* ahead of *k* and L/D; this difference reflects that SHAP measures average absolute marginal contributions, whereas Sobol quantifies variance explained (including interactions). For $y_{M}$, *u* is the largest ($I_{u}\approx 20.7$), followed by *k* ($I_{k}\approx 14.7$) and L/D ($I_{L/D}\approx 8.3$); here too the SHAP ranking (u>k>L/D) differs from the Sobol total-effect order (u>L/D>k).

### 6.3. Partial Dependence Plots

To visualize the marginal effect of one input while averaging over the remaining variables, the partial dependence function is defined as

(53) $${\mathrm{PD}}_{Y,j}\left(x_{j}\right)=E_{x_{∼j}}\;\left[f\left(x\right)|x_{j}\right]\approx \frac{1}{N_{p}}\sum_{n=1}^{N_{p}}f\;\left(x_{j},\;x_{∼j}^{\left(n\right)}\right),$$

where f(·) is the MLP surrogate and $\left\{x_{∼j}^{\left(n\right)}\right\}$ are random samples of the complementary inputs. In this study, each input is discretized with 60 uniformly spaced grid points over the GITT dataset range, while $N_{p}=800$ background samples are obtained by resampling rows from the GITT dataset. For ${\mathrm{PD}}_{\cdot ,u}$ and ${\mathrm{PD}}_{\cdot ,L/D}$, the background is restricted to the same sweep structure as Figure 4 and Figure 5 (L/D=50 for *u*-sweeps and u=3.0 for L/D-sweeps, respectively); for ${\mathrm{PD}}_{\cdot ,k}$, the full dataset is used. At each grid point, background samples are kept only when the varied input lies within the *u*- or L/D-range of the corresponding GITT sweep block (avoiding extrapolation beyond the critical velocity of each *k*); for *k*, the eight discrete values in the dataset are used directly as the grid. The MLP is evaluated $N_{p}$ times per grid point and the output mean (and 5–95% quantile band) is recorded. The shaded band quantifies the spread of individual MLP predictions over the background realizations at fixed $x_{j}$, not a confidence interval for the mean PDP curve; minor wiggles in the *u*- and L/D-panels can appear because the admissible background set changes across GITT sweep blocks, whereas the *k*-panels use only the eight discrete training values and are therefore smoother.

**Figure 9:**
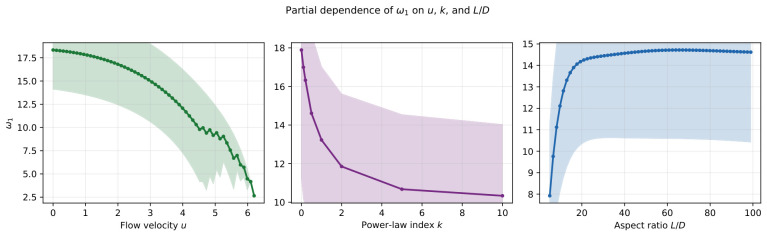
Partial dependence plots of $\omega_{1}$ with respect to *u*, *k*, and L/D. Solid lines denote ${\mathrm{PD}}_{\omega_{1},j}$; shaded bands indicate the 5–95% quantile range of MLP outputs over $N_{p}=800$ background samples (output scatter, not a mean confidence interval).

The partial dependence of $\omega_{1}$ is plotted in Figure 9.

Figure 9 shows that ${\mathrm{PD}}_{\omega_{1},u}\left(u\right)$ decreases monotonically as the flow velocity increases, reproducing the destabilizing effect observed in Figure 4a. The curve steepens at high *u*, where the fundamental frequency approaches the pre-critical regime. The partial dependence on *k* is weak, confirming that $\omega_{1}$ is primarily controlled by *u* and L/D rather than by the gradation index alone. The ${\mathrm{PD}}_{\omega_{1},L/D}\left(L/D\right)$ curve rises from low values at small aspect ratios and flattens beyond L/D≈25–40, matching the Timoshenko-induced plateau in Figure 5a.

**Figure 10:**
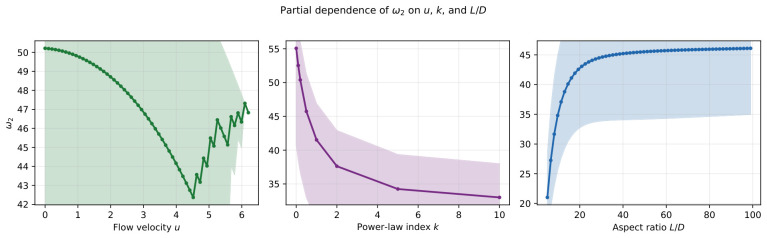
Partial dependence plots of $\omega_{2}$ with respect to *u*, *k*, and L/D. Solid lines denote ${\mathrm{PD}}_{\omega_{2},j}$; shaded bands indicate the 5–95% quantile range of MLP outputs over $N_{p}=800$ background samples (output scatter, not a mean confidence interval).

The partial dependence of $\omega_{2}$ is plotted in Figure 10.

Figure 10 displays qualitatively similar trends for the second mode: $\omega_{2}$ decreases with *u*, shows a stronger dependence on *k* than $\omega_{1}$, and rises at small L/D before converging to a nearly constant value at large aspect ratios. Compared with $\omega_{1}$, the partial dependence on *k* is more pronounced, which is consistent with the higher first-order Sobol index $S_{k}$ in Figure 8 and the larger $I_{k}$ in Table 9.

**Figure 11:**
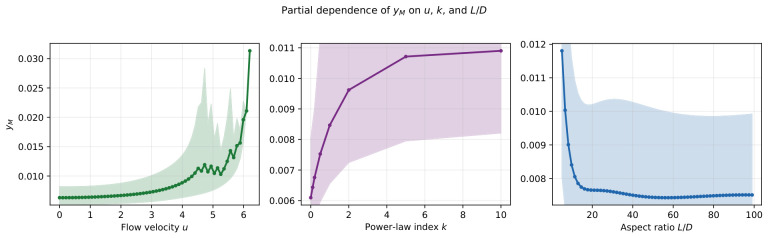
Partial dependence plots of $y_{M}$ with respect to *u*, *k*, and L/D. Solid lines denote ${\mathrm{PD}}_{y_{M},j}$; shaded bands indicate the 5–95% quantile range of MLP outputs over $N_{p}=800$ background samples (output scatter, not a mean confidence interval).

The partial dependence of $y_{M}$ is plotted in Figure 11.

Figure 11 reveals the strongest nonlinearities. ${\mathrm{PD}}_{y_{M},u}\left(u\right)$ remains near zero at low flow velocities and grows rapidly as *u* approaches the upper sampling limit, reflecting the critical-speed divergence in Figure 4c. Averaged over the full training domain, ${\mathrm{PD}}_{y_{M},k}\left(k\right)$ increases with *k*, which is consistent with the *u*-sweep trend at L/D=50 (Figure 4c) but differs from the decreasing $y_{M}$–*k* relation on the L/D-sweep at u=3.0 (Figure 5c); the domain-averaged PDP should therefore be read together with the conditional GITT sweeps. The ${\mathrm{PD}}_{y_{M},L/D}\left(L/D\right)$ curve decreases at small L/D and reaches a plateau at large aspect ratios, in agreement with Figure 5c. Together, Figure 9, Figure 10 and Figure 11 confirm that the MLP surrogate preserves the one-dimensional parametric trends extracted from GITT, while Sobol and SHAP analyses quantify the relative importance of each input.

### 6.4. Discussion of Sensitivity Results and Physical Interpretation

The Sobol, SHAP, and PDP results are interpreted together with the GITT parametric trends reported in Section 5.1. According to Figure 8, Figure 9, Figure 10 and Figure 11, the dimensionless flow velocity *u* is the primary factor controlling $\omega_{1}$ and $\omega_{2}$, because increasing *u* monotonically reduces both natural frequencies (Figure 4a,b). For $y_{M}$, both *u* and L/D are highly influential according to the Sobol total-effect indices ($S_{T,u}\approx 0.78$, $S_{T,L/D}\approx 0.61$), while the SHAP ranking gives u>k>L/D (Table 9). The maximum deflection grows rapidly as *u* approaches the critical velocity (Figure 4c and Figure 11), while the aspect ratio controls the stiffness-related plateau observed in Figure 5c. The power-law index *k* modulates the effective stiffness distribution and therefore contributes more noticeably to $\omega_{2}$ and $y_{M}$ than to $\omega_{1}$ ($S_{T,k}\approx 0.25$ and 0.33 versus 0.02), although *u* and L/D generally remain the leading Sobol factors within the studied parameter ranges. The close agreement between the PDP curves and the GITT reference sweeps validates that the surrogate captures not only pointwise accuracy (Section 5.2) but also the global input–output structure required for design-oriented sensitivity studies.

## 7. Conclusions

This study investigated the vibration characteristics of clamped–clamped Alumina–Steel axially functionally graded (AFG) fluid-conveying Timoshenko pipes using a physics-based GITT benchmark and an MLP surrogate trained on GITT data. The key novelty is the systematic coupling of high-fidelity GITT solutions with a lightweight feedforward surrogate within a single workflow, bridging the gap between physics-based benchmarking and the computational speed required for iterative design. The workflow couples high-fidelity GITT solutions, a lightweight feedforward surrogate, and downstream global sensitivity analysis that would be computationally prohibitive if each query required a full Fortran GITT run. The main findings are summarized as follows.

First, the Timoshenko–GITT benchmark extended to an Alumina–Steel AFG pipe provides a unified high-fidelity reference for the coupled influence of the power-law index *k*, dimensionless flow velocity *u*, and aspect ratio L/D on the first two natural frequencies ($\omega_{1}$, $\omega_{2}$) and the maximum vibration deflection ($y_{M}$). The parametric results show that increasing *k* reduces $\omega_{1}$ and $\omega_{2}$; on *u*-sweeps at L/D=50, larger *k* also increases $y_{M}$ and lowers the critical flow velocity, whereas on L/D-sweeps at u=3.0 the maximum deflection decreases with *k*. Higher flow velocity destabilizes the system, and smaller aspect ratios make Timoshenko shear effects more important.

Second, a feedforward MLP surrogate is developed from $N_{s}=336$ GITT benchmark samples. To respect the ordered structure of the parametric sweeps, an interior block-wise 80/20 train–test split ($N_{\mathrm{tr}}=270$, $N_{\mathrm{te}}=66$) is adopted, and three independent networks with output-specific preprocessing (including a logarithmic transform for $y_{M}$) are trained to map (k,u,L/D) to $\left(\omega_{1},\omega_{2},y_{M}\right)$. On the independent test set, the surrogate achieves $R^{2}=0.9982$, 0.9995, and 0.9911, with $\varepsilon_{\max}=8.90\%$, 2.25%, and 8.89%, respectively; on the training set, the corresponding $R^{2}$ values are 0.9983, 0.9995, and 0.9974. Curve-overlay validation further shows that all four representative GITT sweeps are reproduced with $\varepsilon_{\mathrm{overlay},\max}<9\%$, confirming that the metamodel preserves parametric trends rather than only isolated pointwise accuracy.

Third, global sensitivity analysis based on Sobol indices, SHAP values, and partial dependence plots is performed on the trained surrogate to quantify input importance at a cost affordable only because MLP inference replaces repeated GITT launches. Sobol analysis identifies *u* as the leading total-effect factor for $\omega_{1}$ ($S_{T,u}\approx 0.84$) and as the largest total-effect contributor for $\omega_{2}$ and $y_{M}$ ($S_{T,u}\approx 0.53$ and 0.78); SHAP additionally ranks *k* first for $\omega_{2}$ ($I_{k}\approx 6.94$). For $y_{M}$, Sobol indices rank *u* and L/D ahead of *k* ($S_{T,L/D}\approx 0.61$), whereas SHAP gives u>k>L/D; both methods agree that *u* is primary. The PDP curves reproduce the GITT parametric trends, demonstrating that the surrogate retains the physical input–output structure needed for design-oriented interpretation.

Fourth, timing comparison shows that one online Fortran GITT query requires about $T_{\mathrm{GITT}}=47$ s on average (122 s for L/D=50), whereas a single MLP forward pass requires only $T_{\mathrm{MLP}}=0.085$ ms after one-time offline training ($T_{\mathrm{train}}\approx 0.77$ s), yielding single-query speedups of order $10^{5}$. For $N_{q}=10^{4}$ parametric evaluations, repeated GITT runs require about 131 h, whereas the trained MLP completes the same sweep in about 1.6 s ($\eta \approx 2.9\times 10^{5}$). By reducing the cost of $10^{4}$ parametric evaluations from approximately 131 h to about 1.6 s, the physics-based GITT and MLP surrogate workflow makes real-time design optimization, reliability assessment, Monte Carlo simulation, and sensitivity-driven material screening practically feasible for Alumina–Steel AFG fluid-conveying pipes. These tasks would otherwise be computationally prohibitive with repeated high-fidelity GITT runs alone.

Future work will extend the surrogate to additional boundary conditions, higher-order modes, and broader parameter ranges, and will incorporate uncertainty quantification for predictions outside the original training domain.

## Figures and Tables

**Table 1:** Comparison between the GITT benchmark solver and the MLP surrogate model.

Item	GITT Benchmark	MLP Surrogate
Role in this study	High-fidelity physics-based solver	Data-driven metamodel for rapid evaluation
Governing information	Full Timoshenko PDEs + integral transform	Input–output mapping learned from GITT data
Inputs	*k*, *u*, L/D	*k*, *u*, L/D
Outputs	$\omega_{1}$, $\omega_{2}$, $y_{M}$	ω^1, ω^2, y^M
Main computational tasks	Eigenfunctions, coefficient integrals, ODE integration	Forward propagation of trained networks
Typical use	Benchmark generation, transient/spectral analysis	Parametric sweeps, optimization, sensitivity analysis
Accuracy target	Reference solution	Match GITT on independent test set ($R^{2}>0.99$, $\varepsilon_{\max}<9\%$)
Computational cost	High per query	Low per query after one-time training

**Table 2:** Physical properties and parameters.

Parameter	Symbol	Unit	Numerical Value
Pipe outer diameter	*D*	m	0.03
Pipe inner diameter	*d*	m	0.026
Pipe length	*L*	m	5–100*D*
Aspect ratio	L/D	–	5–100
Fluid density	$\rho_{w}$	kg/m^3^	1000
Alumina density	$\rho_{L}$	kg/m^3^	3960
Steel density	$\rho_{R}$	kg/m^3^	7800
Alumina Young’s modulus	$E_{L}$	GPa	390
Steel Young’s modulus	$E_{R}$	GPa	210
Dimensionless flow velocity	*u*	–	0–6.2 (2π critical scale)

**Table 3:** MLP surrogate accuracy on the independent test set ($N_{\mathrm{te}}=66$).

Output	$R^{2}$	MAE	RMSE	$\varepsilon_{\max}$ (%)	$\varepsilon_{\mathrm{mean}}$ (%)
$\omega_{1}$	0.9982	0.1490	0.1970	8.90	1.50
$\omega_{2}$	0.9995	0.1912	0.2456	2.25	0.49
$y_{M}$	0.9911	$1.70\times 10^{-4}$	$3.34\times 10^{-4}$	8.89	1.45

**Table 4:** MLP surrogate accuracy on the training set ($N_{\mathrm{tr}}=270$, reference).

Output	$R^{2}$	MAE	RMSE	$\varepsilon_{\max}$ (%)	$\varepsilon_{\mathrm{mean}}$ (%)
$\omega_{1}$	0.9983	0.1389	0.1808	8.86	1.21
$\omega_{2}$	0.9995	0.1527	0.2083	2.95	0.39
$y_{M}$	0.9974	$9.42\times 10^{-5}$	$1.98\times 10^{-4}$	7.02	0.93

**Table 5:** Summary of the MLP surrogate model formulation.

Item	Definition
Input vector	$x={\left[u,\;k,\;L/D\right]}^{T}$
Output vector	$y={\left[\omega_{1},\;\omega_{2},\;y_{M}\right]}^{T}$
Total GITT samples	$N_{s}=336$
Training/test split	$N_{\mathrm{tr}}=270$/$N_{\mathrm{te}}=66$ (80/20, interior block-wise)
Network type	Three independent feedforward MLPs
Hidden layers	45, 25 neurons
Hidden activation	tanh(·), Equation (31)
Output activation	Linear, Equation (32)
Output transform	${\overset{ˇ}{y}}_{3}=\ln\left(y_{M}\right)$, Equation (27)
Loss function	MSE, Equation (34)
Training algorithm	L-BFGS (lbfgs, scikit-learn)
Validation	Section 5.2, Table 3 and Table 4, Equayions (40)–(44)

**Table 6:** Maximum relative error between GITT curves and MLP reconstructions for selected parametric sweeps.

Parametric Sweep	GITT Figure	$\varepsilon_{\mathrm{overlay},\max}$ (%)
$\omega_{1}$ vs. *u* (L/D=50)	Figure 4a	8.90
$\omega_{2}$ vs. *u* (L/D=50)	Figure 4b	1.14
$y_{M}$ vs. *u* (L/D=50)	Figure 4c	8.89
$y_{M}$ vs. L/D (u=3.0)	Figure 5c	5.44

**Table 7:** Wall-clock time per Fortran GITT query during *u*-sweeps at k=10 (from intervals between consecutive output files).

L/D	$N_{u}$	Total Sweep Time	$T_{\mathrm{GITT}}$ (s)
5	30	5 min	10
10	31	7 min	13
20	31	17 min	33
30	31	29 min	56
50	31	63 min	122
Mean over L/D=5, 10, 20, 30, 50			47

**Table 8:** Computational time comparison between online Fortran GITT runs and MLP inference (GITT: mean over Table 7; MLP: Python workstation). After a one-time offline training of only $T_{\mathrm{train}}=0.77$ s, the trained MLP delivers single-query inference in $T_{\mathrm{MLP}}=0.085$ ms, yielding a speedup factor $\eta \approx 2.9\times 10^{5}$ for $N_{q}=10^{4}$ parametric evaluations.

Evaluation Strategy	Single-Query Time	One-Time Offline Cost	$N_{q}=10^{4}$ Parametric Sweep
GITT (online)	$T_{\mathrm{GITT}}=47$ s	–	$N_{q}T_{\mathrm{GITT}}=4.7\times 10^{5}$ s (≈131 h)
MLP (inference only)	$T_{\mathrm{MLP}}=0.085$ ms	$T_{\mathrm{train}}=0.77$ s	$T_{\mathrm{train}}+N_{q}T_{\mathrm{MLP}}=1.6$ s
Speedup factor η	$5.5\times 10^{5}$	–	$2.9\times 10^{5}$

**Table 9:** Mean absolute SHAP values ($I_{j}=E[|\phi_{j}\left|\right]$) for each output. Values are computed by KernelSHAP on the trained MLP; within each row, larger $I_{j}$ indicates stronger global influence.

Output	$I_{u}$	$I_{k}$	$I_{L/D}$
$\omega_{1}$	5.06	2.85	2.75
$\omega_{2}$	2.56	6.94	3.43
$y_{M}$	20.70	14.66	8.27

## Data Availability

The original contributions presented in this study are included in the article. Further inquiries can be directed to the corresponding author.
